# A recurrent *SHANK3* frameshift variant in Autism Spectrum Disorder

**DOI:** 10.1038/s41525-021-00254-0

**Published:** 2021-11-04

**Authors:** Livia O. Loureiro, Jennifer L. Howe, Miriam S. Reuter, Alana Iaboni, Kristina Calli, Delnaz Roshandel, Iva Pritišanac, Alan Moses, Julie D. Forman-Kay, Brett Trost, Mehdi Zarrei, Olivia Rennie, Lynette Y. S. Lau, Christian R. Marshall, Siddharth Srivastava, Brianna Godlewski, Elizabeth D. Buttermore, Mustafa Sahin, Dean Hartley, Thomas Frazier, Jacob Vorstman, Stelios Georgiades, Suzanne M. E. Lewis, Peter Szatmari, Clarrisa A. (Lisa) Bradley, Anne-Claude Tabet, Marjolaine Willems, Serge Lumbroso, Amélie Piton, James Lespinasse, Richard Delorme, Thomas Bourgeron, Evdokia Anagnostou, Stephen W. Scherer

**Affiliations:** 1 Genetics and Genome Biology and The Centre for Applied Genomics, The Hospital for Sick Children, Toronto, ON Canada; 2 Canada’s Genomics Enterprise (CGEn), The Hospital for Sick Children, Toronto, ON Canada; 3 Holland Bloorview Kids Rehabilitation Hospital, Toronto, ON Canada; 4 Department of Medical Genetics, BC Children’s Hospital Research Institute, University of British Columbia, Vancouver, BC Canada; 5 Program in Molecular Medicine, The Hospital for Sick Children, Toronto, ON Canada; 6 Department of Cell & Systems Biology, University of Toronto, Toronto, ON Canada; 7 Department of Biochemistry, University of Toronto, Toronto, ON Canada; 8 Genome Diagnostics, Department of Paediatric Laboratory Medicine, The Hospital for Sick Children, Toronto, ON Canada; 9 Department of Laboratory Medicine and Pathobiology, University of Toronto, Toronto, ON Canada; 10 Department of Neurology, Rosamund Stone Zander Translational Neuroscience Center, Boston Children’s Hospital, Harvard Medical School, Boston, MA USA; 11 Autism Speaks, New York, NY USA; 12 Autism Speaks and Department of Psychology, John Carroll University, Cleveland, OH USA; 13 Department of Psychiatry, University of Toronto, Toronto, ON Canada; 14 Department of Psychiatry, The Hospital for Sick Children, Toronto, ON Canada; 15 Department of Psychiatry and Behavioural Neurosciences, McMaster University, Hamilton, ON Canada; 16 Centre for Addiction and Mental Health, Toronto, ON Canada; 17 Human Genetics and Cognitive Functions, Institut Pasteur, UMR3571 CNRS, Université de Paris, F-75015 Paris, France; 18 Genetics Department, Cytogenetic Unit, Robert Debré Hospital, APHP, F-75019 Paris, France; 19 Service de Génétique clinique, CH de Chambéry, Chambéry, France; 20 Biochimie et Biologie Moléculaire, CHU Nimes, Univ. Montpellier, Nimes, France; 21 Institut de Génétique et de Biologie Moléculaire et Cellulaire, Centre National de la Recherche Scientifique, UMR7104, Institut National de la Santé et de la Recherche Médicale, U964, Université de Strasbourg, Illkirch, France; 22 Unité de Génétique Moléculaire, IGMA, Hôpitaux Universitaire de Strasbourg, Strasbourg, France; 23 Institut Universitaire de France, Paris, France; 24 Child and Adolescent Psychiatry Department, Robert Debré Hospital, APHP, F-75019 Paris, France; 25 Department of Paediatrics, University of Toronto, Toronto, ON Canada; 26 Department of Molecular Genetics and the McLaughlin Centre, University of Toronto, Toronto, ON Canada

**Keywords:** Medical genomics

## Abstract

Autism Spectrum Disorder (ASD) is genetically complex with ~100 copy number variants and genes involved. To try to establish more definitive genotype and phenotype correlations in ASD, we searched genome sequence data, and the literature, for recurrent predicted damaging sequence-level variants affecting single genes. We identified 18 individuals from 16 unrelated families carrying a heterozygous guanine duplication (c.3679dup; p.Ala1227Glyfs*69) occurring within a string of 8 guanines (genomic location [hg38]g.50,721,512dup) affecting *SHANK3*, a prototypical ASD gene (0.08% of ASD-affected individuals carried the predicted p.Ala1227Glyfs*69 frameshift variant). Most probands carried *de novo* mutations, but five individuals in three families inherited it through somatic mosaicism. We scrutinized the phenotype of p.Ala1227Glyfs*69 carriers, and while everyone (17/17) formally tested for ASD carried a diagnosis, there was the variable expression of core ASD features both within and between families. Defining such recurrent mutational mechanisms underlying an ASD outcome is important for genetic counseling and early intervention.

## Introduction

Autism Spectrum Disorder (ASD) is a heterogeneous condition, both in clinical presentation and in terms of the underlying etiology. Individuals with ASD are increasingly being seen in clinical genetics^1,2^. More than 100 genetic disorders that can exhibit features of ASD (e.g., Fragile X, Phelan-McDermid syndromes, Rett)^3^ and dozens of rare susceptibility genes (e.g., *NLGN*, *NRXN*, *SHANK* family genes), and copy number variation (CNV) loci (e.g., 1q21.1 duplication,15q11-q13 duplication, 16p11.2 deletion), have been identified, which combined can facilitate a molecular diagnosis in ~5–40% of ASD cases^4–7^. The likelihood of a genetic finding in ASD is dependent on the complexity of the phenotype (e.g., idiopathic or syndromic, with or without intellectual disability)^8,9^, the genomic technology used (e.g., microarrays, exome sequencing, genome sequencing, or combinations thereof)^10^, as well as the annotation pipeline and “gene lists” used for interpretation^11,12^.

There are examples of how understanding the genetic subtypes of ASD can assist early identification enabling earlier behavioral intervention, and informing prognosis, medical management, and assessment of familial recurrence risk^13,14^. Moreover, genomic data promise to facilitate pharmacologic-intervention trials through stratification based on pathway profiles^15,16^. To support these applications, there is a growing interest in performing robust genetic analyses, often in families and in unique populations, linked to deep phenotyping^17–19^.

The largest datasets available for genotype/phenotype correlations in ASD studies are based on CNV assessment since microarrays became the first-tier clinical diagnostic test^20,21^. The most relevant finding from this vast literature is that even for recurrent CNVs (i.e., genomic disorders) involved in ASD, which typically affect the same genes, there is the variable expression of phenotypes relevant to the core features in autism, and other medical features^22–25^.

More recently, genotype and phenotype studies of sequence-level variation (single-nucleotide variants, or SNV, and insertion/deletion, or indel events) affecting individual genes are starting to reveal clinical correlations in ASD. For example, loss-of-function variants in the *SCN2A* sodium channel gene impair glutamatergic neuronal excitability, leading to ASD and/or intellectual disability, while gain of function variants potentiate excitability leading to infantile-onset seizure phenotypes^26^. Different germline dominant-acting mutations in the phosphatase and tensin homolog (*PTEN*) gene found in ASD lead to an increased average head circumference in children^27^. Loss-of-function variants in the *CHD8* chromodomain helicase DNA- binding protein eight gene are also found in overgrowth and intellectual disability forms of ASD^28^. Despite some progress in resolving genotype-phenotype correlations, the vast genetic complexity and variable expressivity of genes involved in ASD continue to confound most predictive studies.

Following a genotype-first approach, here we initially searched available ASD-specific, controlled access, genome-wide sequence databases, such as MSSNG (https://research.mss.ng) and Simon’s Simplex Collection (SSC) (https://www.sfari.org/resource/sfari-base) as well as our own in-house data (available in the next MSSNG data release) to identify recurrent sequence-level damaging variants (*de novo* loss-of function or missense variants predicted to be damaging based on the American College of Medical Genetics guidelines^29^) affecting the same site (genomic location) in the same gene in different families. The database searches were then followed by a literature survey to identify additional individuals reported to have the same variant. In our most compelling finding, we identified a mutational ‘hotspot’ in a string of 8-Gs in exon 21 (p.Ala1227Glyfs*69) of the *SHANK3* gene that was present in 17 individuals from 15 unrelated families with ASD, as well as one individual with several autistic features and Phelan-McDermid Syndrome (but who was not tested for ASD). The individuals identified in both the ASD-specific databases and the published manuscripts had various details available describing the phenotype which we have summarized. We were able to contact the families that are described for the first time in this paper to gather additional information. Using these available data, we assessed the intra- and inter-familial phenotypic variation (as well as all other genetic information) within these individuals and discuss the findings in the context of genotype-phenotype comparison, including variable expression of ASD core symptom and related features.

## Results

### Identification of the recurrent p.Ala1227Glyfs*69 variant

To achieve the most comprehensive genomic representation (difficult to sequence exons, splice site boundaries) for variant detection, we initially examined the Autism Speaks MSSNG whole-genome sequencing (WGS) cohort (https://research.mss.ng/), with 11,359 samples, including 5102 affected individuals and 3567 with family data, typically belonging to trios, or quads (two parents and two affected children) for recurrent mutations. Secondly, we tested the Simon Simplex Collection (SSC) WGS collection (https://www.sfari.org/resource/simons-simplex-collection/), which comprises 9,205 samples, including 2419 affected individuals and 2393 with family data (typically two parents, one affected child, one unaffected child). Previous studies have extensively reported on MSSNG^6,17,30,31^ and SSC^32,33^. Probands from both cohorts met the criteria for ASD based on scores from standardized diagnostic criteria tools, typically the Autism Diagnostic Observation Schedule (ADOS)^34^ and the Autism Diagnostic Interview–Revised (ADI-R)^35^ and/or was supported by clinical criteria. Many individuals were also assessed with standardized measures of intelligence (I.Q.), including verbal and nonverbal ability, language, social behavior, adaptive functioning, and physical measurements^6,32,33^. All of this phenotype data is available from the respective databases.

From the genome sequences analyzed, our most interesting finding identified five probands in MSSNG (four males and one female) from four families and one proband in SSC (male) carrying a heterozygous guanine duplication in *SHANK3* (NCBI: NM_033517.1; ENSEMBL: ENST00000262795.5; c.3679 or c.3676 depending on the transcript) (Table 1; the reference sequence NM_033517.1 was selected as the appropriate transcript for this study as this was the reference sequence used in the original publication of this variant in Durand et al.^36^). We also found other recurrent sequence-level *de novo* heterozygous damaging missense variants in the *PTEN*, *CAMK2A*, *SPTAN1*, *MECP2*, and *CSNK1E* genes, but in each of these instances no more than two unrelated individuals were found in the combined MSSNG and SSC data (Supplementary Material; Table S1).

**Table 1:** Genome annotation of the *SHANK3* guanine duplication (rs797044936).

Reference genome	Transcript accession	Exon	Genomic position	Coding change	Protein change	Annotation tool
Hg38	NM_033517.1	21	Chr22:50721512–50721513	c.3679dup	p.(Ala1227Glyfs*69)	^1^Alamut Visual v2.15.0
	^2^NM_001080420.1	22	Chr22:g.50721512dup	c.3727dup	p.(Ala1243Glyfs*69)	Alamut Visual v2.15.0
	ENST00000262795.5	24	Chr22:g.50721512dup	c.3676dup	p.(Ala1226Glyfs*69)	Alamut Visual v2.15.0
	NM_001372044 (replaced NM_033517)	22	22-50721503-50721504-T-TG	c.3855dupG	p.L1285fs	MSSNG
Hg19	NM_033517.1	21	Chr22:51159940–51159941	c.3679dup	p.(Ala1227Glyfs*69)	Alamut Visual v2.15.0
	NM_001080420.1	22	Chr22:g.51159940dup	c.3727dup	p.(Ala1243Glyfs*69)	Alamut Visual v2.15.0
	ENST00000262795.5	24	Chr22:g.51159940dup	c.3676dup	p.(Ala1226Glyfs*69)	Alamut Visual v2.15.0
	NM_033517	21	chr22:51159932-51159932-T-TG	c.3630dup	p.L1210fs	VariCarta; GATK VariCarta
			chr22:51159933-51159933-G-GG			
	ENST00000262795.3	22	22-5119932-T-TG	c.3719_3720 insG	p.Ala1243GlyfsTer6	O’Roak et al.^40^.
	ENST00000262795.3	22	22-5119932-T-TG	c.3720dupG	p.L1240fs	Feliciano et al.^41^.

The annotation considers different reference genomes, the position of the duplication in the guanine string, and the annotation tool. The guanine duplication in each carrier in the main text of the paper is referred to as p.Ala1227Glyfs*69.

^1^Alamut Visual version 2.15 (SOPHiA GENETICS, Lausanne, Switzerland). This tool annotates the final G as duplicated and provides the coding and protein change as well as ClinVar entries and general population frequencies (Supplementary Fig. S3).

^2^NM_001080420.1 record has been removed from NCBI. This RefSeq was permanently suppressed because currently there is insufficient support for the transcript and the protein. Exon 11 was based on ab initio prediction and is not supported by transcript data.

At the time of submission, the most recent RefSeq NM_01372044, which replaces and updates NM_033517, was not available in the Alamut software.

**Fig. 1:**
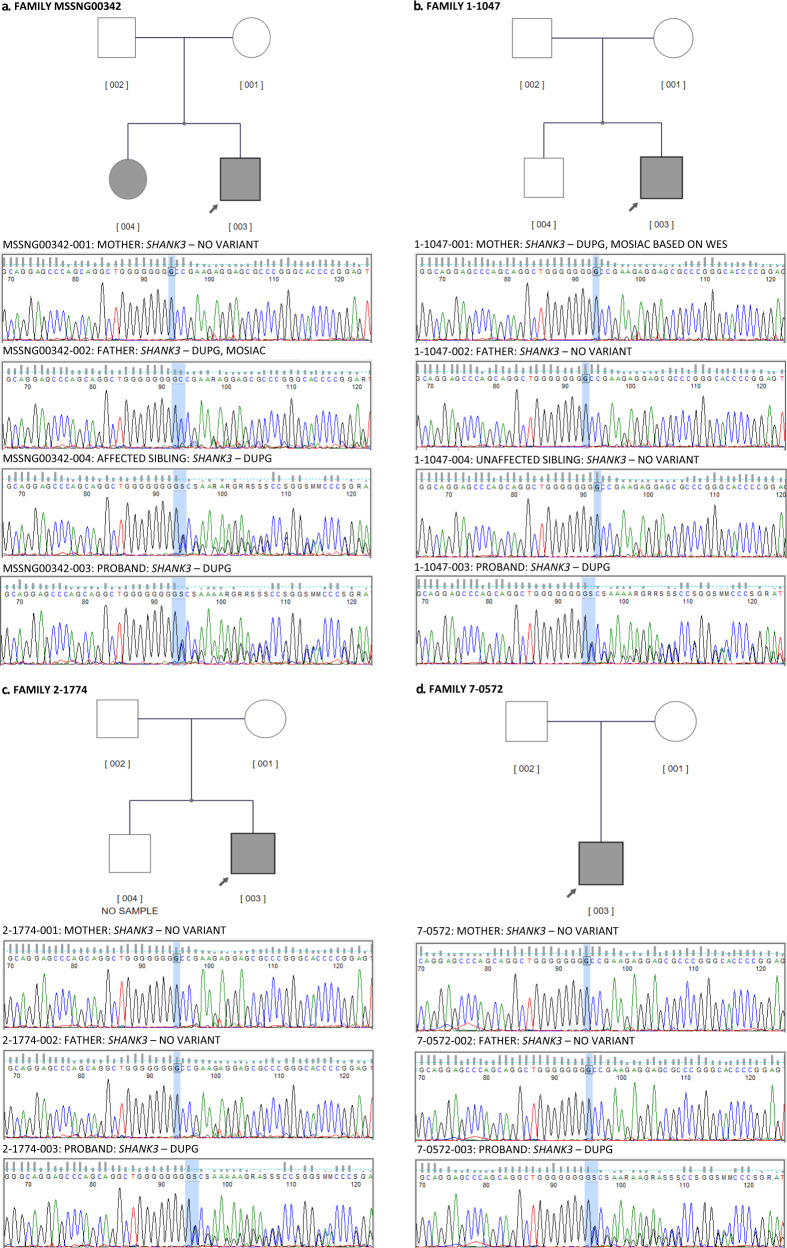
Pedigrees of MSSNG families reported for the first time in this study and their Sanger sequencing confirmation. **A** Pedigree MSSNG00342; **B** Pedigree 1–1047 (unaffected sibling was targeted Sanger sequenced but was not the whole-genome sequenced); **C** Pedigree 2-1774 (unaffected sibling sample was not available); **D**. Pedigree 7–0574 (will be available in MSSNG DB7). Gray shapes indicate individuals with an ASD diagnosis and carry the *SHANK3* variant.

**Fig. 2:**
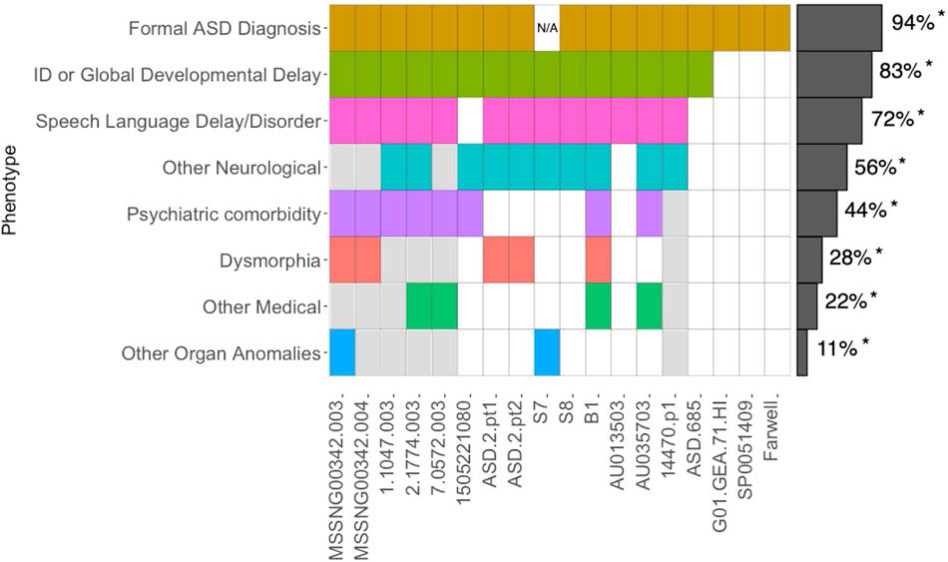
Phenotypic heterogeneity in individuals (X-axis) carrying the *SHANK3* p.Ala1227Glyfs*69 variant reported in the MSSNG^6^, SSC^32,33^, and in published papers^4,36,38–42,71^. Those individuals in the same family are grouped within the black boxes. Gray spaces indicate the absence of the phenotype. White spaces indicate that the phenotype might have not been accessed in the proband. Phenotypic categories are described in Table 3. Individual S7 was not formally reported as being formally tested for ASD. *Caution is needed in the interpretation of these frequencies since some phenotypes were not assessed for some individuals.

The discovery of this recurrent guanine duplication variant in *SHANK3* was confirmed using Sanger sequencing (Fig. 1). We then scanned the literature, including using Varicarta^37^ and found that this same guanine duplication was reported in 12 probands affected by ASD^4,36,38–42^, and one proband within the ASD borderline range, Phelan-McDermid syndrome, significantly delayed language, and speech and visual-motor deficits^38^. We carefully examined all genotypes and found that one was the same individual in the SSC cohort (14470.p1);^40^ therefore, we removed this duplicate individual. Considering the new cases reported here and the cases reported in the literature, the p.Ala1227Glyfs*69 variant has been observed in a total of 18 cases from 16 families, identified using different genome-testing approaches (Table 2). Nearly all of these probands (17/18) were ascertained for ASD, although the general phenotype, as discussed below, varies somewhat among individuals (Table 3; Fig. 2). We also detected one female individual with ASD (with mild intellectual disability) carrying a *de novo* G deletion (7-G’s) at this same site (c.3679del p.Ala1227Profs*57).

**Table 2:** ASD probands identified in MSSNG, SSC, and other publications containing the p.Ala1227Glyfs*69 *SHANK3* variant.

ID	SEX	GENOMIC (H.G.)	REFERENCE SEQUENCE	CODING	PROTEIN	INHERITANCE	PUBLICATION/ COHORT	ANCESTRY	TECHNOLOGY	^1^PRS
MSSNG00342-003	M	g.50721512dup (hg38)	NM_033517.1	c.3679dup	p.A1227Gfs*69	Paternal, mosaic	MSSNG—This paper	European	WGS	3.639
MSSNG00342-004	F	g.50721512dup (hg38)	NM_033517.1	c.3679dup	p.A1227Gfs*69	Paternal, mosaic	MSSNG—This paper	European	WGS	6.336
1-1047-003	M	g.50721512dup (hg38)	NM_033517.1	c.3679dup	p.A1227Gfs*69	Maternal, mosaic	MSSNG—This paper	European	WGS	−1.167
2-1774-003	M	g.50721512dup (hg38)	NM_033517.1	c.3679dup	p.A1227Gfs*69	*De novo*	MSSNG—This paper	European	WGS	7.035
7-0572-003	M	g.50721512dup (hg38)	NM_033517.1	c.3679dup	p.A1227Gfs*69	*De novo*	MSSNG-DB7—This paper	European	WGS	15.606
1505221080	M	g.50721512dup (hg38)	NM_033517.1	c.3679dup	p.A1227Gfs*69	*De novo*	This paper	N/A	Direct Sanger sequencing	Not estimated
HNDS_0130-01	F	g.50721512dup (hg38)	NM_033517.1	c.3679dupG	p.A1227Gfs*69	*De novo*	This paper	N/A	WES	Not estimated
ASD-2 pt1	M		NM_033517.1	c.3679dup		Maternal, mosaic	Durand et al.^37^.	European	FISH and direct sequencing	Not estimated
ASD-2 pt2	M		NM_033517.1	c.3679dup		Maternal, mosaic	Durand et al.^37^.	N/A	FISH and direct sequencing	Not estimated
S7	F	g.51159940dupG (hg19)	NM_033517.1	c.3679dupG	p.A1227Gfs*69	Non-paternal	De Rubeis et al.^38^.	N/A	WES	Not estimated
S8	M	g.51159940dupG (hg19)	NM_033517.1	c.3679dupG	p.A1227Gfs*69	*De novo*	De Rubeis et al.^38^.	N/A	WES	Not estimated
B1	F	g.51159940dupG (hg19)	NM_033517.1	c.3679dupG	p.A1227Gfs*69	*De novo*	De Rubeis et al.^38^.	N/A	WES	Not estimated
AU013503	F	(hg19)	NM_033517.1	c.3679dupG	p.Ala1227fs	*De novo* or father	Zhou et al.^39^.	Chinese	Target sequencing	Not estimated
AU035703	F	(hg19)	NM_033517.1	c.3679dupG	p.Ala1227fs	*De novo*	Zhou et al.^39^.	Chinese	Target sequencing	Not estimated
14470.p1	M	22-51159932 -T-TG(hg19)	ENST00000262795.3	c.3719_3720insG	p.Ala1243GlyfsTer6	*De novo*	O’Roak et al.^40^.	European	WES - O’Roak et al. 2014 WGS – This paper	6.718
ASD-685	M		NM_033517.1	c.3630dupG	p.L1210fs	*De novo*	Du et al.^71^.	Chinese	WES	Not estimated
G01-GEA-71-HI	F	22:51159932:T:TG (hg19)				*De novo*	Satterstrom et al.^4^.	N/A	WES	Not estimated
SP0051409	F	22- 51159932 -T-TG(hg19)	ENST00000262795.3	c.3720dupG	p.L1240fs	*De novo*	Feliciano et al.^41^.	N/A	WES	Not estimated
Farwell - N/A	N/A	N/A	NM_033517.1	c.3679dupG	p.A1227Gfs*69	*De novo*	Farwell et al.^42^.	American	WES	Not estimated
^2^ClinVar SCV000850848 History of Neurodevelopmental Disorder	N/A	Chr22: g.50721512dup (hg38)	NM_033517.1	c.3679dup	p.Ala1227fs	N/A	Ambry Genetics	N/A	Clinical testing	Not estimated
ClinVar SCV000244220 Inborn genetic diseases	N/A	Chr22: g.50721512dup (hg38)	NM_033517.1	c.3679dup	p.Ala1227fs	N/A	Ambry Genetics	N/A	Clinical testing	Not estimated
ClinVar SCV001149930 22q13.3 deletion syndrome	N/A	Chr22: g.50721512dup (hg38)	NM_033517.1	c.3679dup	p.Ala1227fs	*De novo*	Institute of Human Genetics, Klinikum rechts der Isar	N/A	Clinical testing	Not estimated
ClinVar SCV001149930 22q13.3 deletion syndrome	N/A	Chr22: g.50721512dup (hg38)	NM_033517.1	c.3679dup	p.Ala1227fs	*De novo*	Institute for Genomic Statistics and Bioinformatics, University Hospital Bonn	N/A	Clinical testing	Not estimated
ClinVar SCV000329516 No condition provided	N/A	Chr22: g.50721512dup (hg38)	NM_033517.1	c.3679dup	p.Ala1227fs	N/A	GeneDx	N/A	Clinical testing	Not estimated
ClinVar SCV001468904 No condition provided	N/A	Chr22: g.50721512dup (hg38)	NM_033517.1	c.3679dup	p.Ala1227fs	N/A	Laboratoire de Génétique Moléculaire, CHU Bordeaux	N/A	Clinical testing	Not estimated

Thicker lines box individuals from the same family. *WGS* whole-genome sequencing, *WES* whole-exome sequencing, *PRS* polygenetic risk score, *N/A* not available. Note that individual S7 described in De Rubeis^38^ has not been formally diagnosed with ASD but has reported autism-associated phenotypes. We also detected one female individual with ASD carrying a *de novo* G deletion (7-G’s) at this same site (c.3679del p.Ala1227Profs*57).

^1^For the PRS in addition to the main text only SNPs with minor allele frequency of >0.05 in controls and high imputation quality (INFO >0.9) were included. SNPs within the broad MHC region (Chr6:25–35MB) were excluded as well as all ambiguous SNPs to avoid potential strand conflicts. Only variants with good sequencing quality (filter = PASS) were included. Clumping was done with r^2^ threshold and radius set at 0.1 and 500 kb, respectively. Subsequently, PRS was generated with *p* value threshold of 0.1 weighing by the additive scale effect (log (OR)) of each variant and summing over the variants using PLINK 1.9. The scores were centered by the mean in whole population. ^2^Variants submitted and cataloged by ClinVar (https://www.ncbi.nlm.nih.gov/clinvar/variation/208759/), Accession VCV000208759.8, provided from clinical testing and interpreted as pathogenic.

**Table 3:** Phenotype of ASD probands identified in MSSNG, SSC, and other publications containing the *SHANK3* p.Ala1227Glyfs*69 variant Thicker lines box individuals from the same family.

ID	SEX	ASD	Dysmorphia	Intellectual disability/developmental delay	Other Medical comorbidities	Psychiatric comorbidity	Other Neurological comorbidities	Other Organ anomalies	Language/speech disorder
^1^MSSNG00342-003	M	Yes	Macrocephaly Mandibular prognathism. Malar flattening	Severe ID		Bipolar disorder		Conductive hearing loss	yes
^1^MSSNG00342-004	F	Yes	Macrocephaly Asymmetric facial features (r; Mandibular prognathism; Prominent supraorbital ridge. Abnormal iris pigmentation. Hirsutism. Thick skin texture	Mild ID		Depression with psychotic tendencies, Self-injurious behavior; late regression			yes
^1^1-1047-003	M	Yes		Severe ID		ADHD	Epilepsy		severe
^1^2-1774-003	M	Yes		Severe ID	Food sensitivities, G.I. distress, Eczema, Sleep disturbance	Anxiety	Epilepsy Hypotonia, Hypermobility		Severe, with early language loss
^1^7-0572-003	M	Yes		ID	Lactose intolerance	PICA (compulsive ingestion of inedible matter)			severe
^1^1505221080	M	Yes		Moderate ID		ADHD	Developmental Coordination Disorder		
^2^ASD-2 pt1	M	Yes	large ears and elbow extension limitation	Moderate ID			Neonatal hypotonia		Severe
^2^ASD-2 pt2	M	Yes	Macrocephaly by 9 months of age, followed by slow growth	Severe ID			Epilepsy		severe
^2^S7	F	Not available		Mild ID			Hypotonia, Visuo-motor deficits	Coronary artery fistula	severe
^2^S8	M	Yes		Severe ID			Hypotonia/Dysphagia, Abnormal gait		yes
^2^B1	F	Yes	Preauricular skin tags Finger and toe-tapping MRI: Bilateral T2 hyper-intensities of posterior centrum semiovale	Mild ID	Scoliosis Sleep disturbance	Hyperactivity	Constipation		Mild
^2^AU013503	F	Yes		Developmental delay					yes
^2^AU035703	F	Yes		Developmental delay	Sleep disturbance	Hyperactivity	Abnormal gait Gastrointestinal complaints		Yes, with regression
^1^14470.p1	M	Yes		Severe ID			Epilepsy		Severe
^2^ASD-685	M	Yes		ID					
G01-GEA-71-HI	F	Yes		Data not available					
SP0051409	F	Yes		Data not available					
Farwell - N/A	N/A	Yes		Data not available					

*ID* intellectual disability, *DD* developmental delay, *ADHD* Attention deficit hyperactivity disorder, *N/A* not available.

Note that individual S7 described in De Rubeis et al.^38^. has not been diagnosed with ASD but has some autism-associated phenotypes. Graphical representation of these phenotypes is presented in Fig. 2.

^1^available clinical diagnosis, or scores in clinically significant range

^2^descriptions from original manuscript

### Genome annotation of the p.Ala1227Glyfs*69 variant

**Fig. 3:**
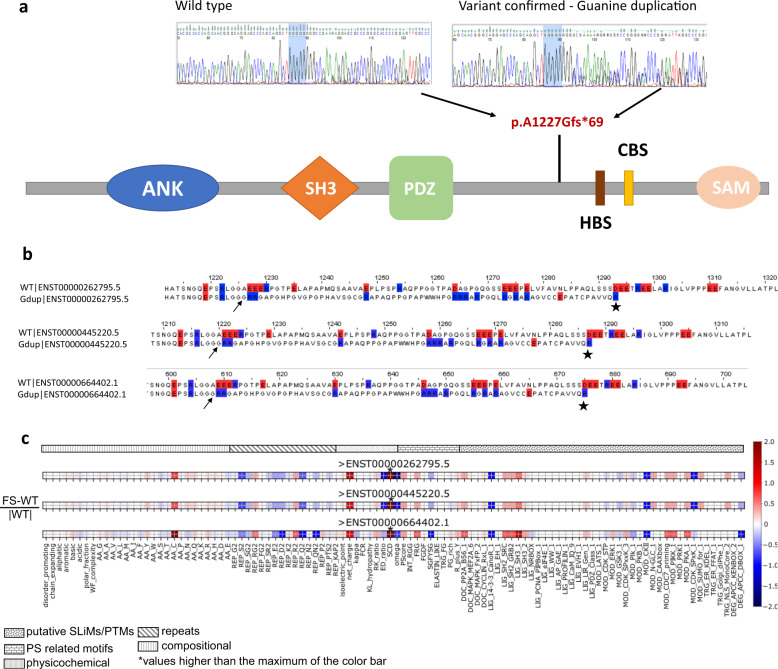
Impact of the *SHANK3* p.Ala1227Glyfs*69 variant on the protein. **A** (top left) Guanine string containing 8 Gs found in non-affected individuals; (top right) Guanine string containing nine Gs found in ASD-affected individuals and parents with somatic mutations; (bottom) Location of the frequent guanine duplication in the *SHANK3* gene. ANK ankyrin repeats, SH3 SRC homology 3 domain, PDZ postsynaptic density 95/Discs large/zona occludens, HBS homer binding site, CBS cortactin binding site, SAM sterile alpha motif domain. **B** Alignment of wild type protein sequences, for each of three highly expressed splice isoforms, to the protein sequence of the variant around the position of the mutation; (note, in this figure the first transcript presented is ENST00000262795.5 and the protein change for this is p.Ala1226Glyfs*69 as shown in Table 1). **C** Normalized impact of the variant for the three isoforms using FAIDR, a tool that identifies physical features and the presence of consensus protein recognition motifs in intrinsically disordered protein regions^56^. (*Note that SCD, sequence charge decoration, a measure of charge patterning associated with phase separation, has values significantly above 2: 5.4, 7.0, and 10.2 for the three isoform.).

The *SHANK3* guanine duplication is located within a segment of 8-G′s on chromosome 22q13 at genomic location [hg38]g.50,721,505dup or g.50,721,512dup, depending on the position that this variant is annotated in the guanines (Table 1; Fig. 2). Some tools annotate the first G as the duplication, and others annotate it as the final G (Supplementary Material; Fig. 3). The sequencing technology might also affect the variant annotation, with Sanger sequencing conventionally adding the G duplication at the 3′ end of the gene as the first point of amino acid change, and Next Generation Sequencing usually left aligning the variant. Independent of the position of the base insertion in the 8-Gs, the frameshift starting in exon 21 results in the new reading frame ending with a stop codon at position 69, causing a truncation lacking the C-terminal region (Fig. 3). We also confirmed that both exome sequencing and WGS reliably captured this 8-G string genomic segment in the short-read sequence (see Methods).

### Segregation and population frequency of the recurrent p.Ala1227Glyfs*69 variant

All the probands identified in this study carried *de novo* variants with the exception of five individuals. One family with two brothers first reported in the initial *SHANK3* ASD-discovery paper^36^ inherited the variant from their mother, who was found to be mosaic. Two siblings within the MSSNG cohort (MSSNG00342-003 and MSSNG00342-004) inherited the variant from their father, who was also shown to be a mosaic (Table 2). In this latter case, the variant was only present in 8 of 50 reads in the father’s WGS data and was verified using a T.A. clone Kit (Invitrogen cat number 45-0046). Proband 1-1047-003 also seems to have inherited the variant from his mother by somatic mosaicism, in whom the variant was present in 1 of 32 reads of the WGS data. Exome sequencing analysis was also performed in this mother, with the variant being observed in 2 of 110 reads. To search for additional potential relevant somatic mutations^43^, we tested the original alignment files in both cohorts using DeNovoGear’s dng-call method for the *SHANK3* locus^44^ using 0.8 as a posterior probability of a *de novo* mutation (ppDNM), but we did not find any other candidates. Considering the families studied in MSSNG and SSC (our most trusted datasets) 6/7,521(0.08%) ASD-affected individuals carried the p.Ala1227Glyfs*69 variant in 5/6,681 (0.07%) of families. The Fisher’s exact test of the association between the frequency in heterozygous individuals in ASD cases and control population databases has a *P* value of 0.029.

### Consequences of p.Ala1227Glyfs*69 on the SHANK3 protein

Nonsense mutations and frameshifts in *SHANK3* can lead to reduced expression, and SHANK3-deficient neurons were found to have an altered phospho-proteome that may explain their decreased dendritic spine density^45^. However, *SHANK3* mRNA is still expressed in truncation mutant-containing induced pluripotent stem cells (iPSCs)^46^ and truncated SHANK3 proteins may have a dominant-negative effects in neurons^47,48^. We therefore explored the consequences of p.Ala1227Glyfs*69 on the SHANK3 protein. We annotated the positions of amino acids to which the variant is mapped according to ENSEMBL and the UCSC genome browser. Using the DISOPRED3 predictor^49^ and the consensus of eight predictors from MobiDB-lite^50^, we identified where the mutation falls with respect to intrinsically disordered regions (IDRs) of the protein, which may influence protein folding and binding^51^. In both predictors, the position of interest was found to be embedded within a large IDR, which map to multiple isoforms (Fig. 3B). Mutations that create frameshifts and stop codons in this region of *SHANK3*^36,52^ truncate two proline-rich binding sites for Homer and Cortactin (Fig. 3A) and affect function, including altering neuronal morphology in cell-based experiments^46,47^. The SHANK3 protein serves as a scaffold to connect membrane receptors to the actin-cytoskeleton in the postsynaptic density (PSD), a protein-rich sub-compartment considered to be a biomolecular condensate formed by phase separation^53,54^ due to multivalent interactions^46^. In each of the isoforms, these truncations are expected to impair canonical PSD formation and stability.

The variant isoforms were also analyzed using Feature Analysis of Intrinsically Disordered Regions, a tool that identifies the presence of consensus protein recognition motifs in IDRs^55,56^ and using PScore^57^, predicts phase separation propensity via IDR planar pi-contacts (Fig. 3C; Supplementary Material; Fig. S2). A number of specific short linear interaction motifs were found to be altered. Of particular interest is the increase in SH3 domain class I-binding motifs, given that *SHANK3* is known to interact with numerous SH3 domains. The variants significantly increase the number of arginine-glycine and arginine-arginine dipeptide instances, which are associated with mRNA binding and phase separation, and increase the cysteine content of the sequence. A reduction in SHANK3 protein due to the frameshift (e.g., through nonsense mediated decay; discussed below) could also affect the phase separation of the PSD, which is known to be concentration dependent^58^.

### p.Ala1227Glyfs*69 as a pathogenic variant

The p.Ala1227Glyfs*69 variant is classified in ClinVar as “Pathogenic for ASD, NDD, and others” and is exceptionally rare or absent in control populations (ClinVar; https://www.ncbi.nlm.nih.gov/clinvar/variation/208759/). In the gnomAD v2.1.1 dataset^59^, which uses the hg37 as reference genome, it has an allele frequency of 16/160,994 alleles = 0.000099 (0.0099%). In ALFA^60^, this variant is also reported in 0.02% of control Europeans samples. However, in gnomAD v3, 1000 Genomes Project (that uses hg38 as a reference genome), TOPMed^61^, two unpublished pediatric controls from our group (INOVA and CHILD), the Personal Genome Project Canada^62^ and Medical Genome Reference Bank^63^ this variant is not present. In combination, this suggests that the presence of the variant in gnomAD v2.1.1 and ALFA might be due to low-quality sequencing with the preliminary description being corrected in gnomAD v3. It is also noteworthy that ~1/100 people will have ASD, so it would be expected to find p.Ala1227Glyfs*69 variant carriers in control populations. Based on our findings described here they would likely have ASD, but additional studies will be required to further assess this.

We have analyzed the genomic conservation of this variant with GERP^64^, UCSC PhyloP, and phastCons for primates, placental mammals, and 100 vertebrates^65^. GERP identifies constrained elements in multiple alignments by quantifying substitution deficits. These deficits represent substitutions that would have occurred if the element were neutral DNA but did not occur because the element has been under functional constraint. The p.Ala1227Glyfs*69 variant has a GERP score of 5.2 (*p* = 0), suggestive of having a large deleterious effect^66^. The PhyloP score was 0.6 for primates, 1.35 for mammals, and 2.13 considering 100 vertebrates, suggesting high evolutionary conservation. The PhastCon scores were also higher than 0.98 for primates, mammals, and vertebrates, which indicates a strong negative selection on this variant.

### Genotype and phenotype correlation

In all 17 p.Ala1227Glyfs*69 carriers evaluated for ASD, ASD was confirmed by review of the ASD gold standard diagnostic tests available in the databases or as reported in the original manuscripts, and the majority of participants described are reported to have an intellectual disability defined as an IQ score below 70 and impairments in adaptive functioning, although the spectrum of severity is wide (Table 3; Fig. 2). Four individuals were ascertained for Phelan-McDermid Syndrome, with three of these being of the 17 receiving a formal ASD diagnosis and one never being assessed for autism. Language deficits are also prevalent and often severe. We were cautious about making claims on other associated conditions as they have not been universally and systematically ascertained. However, hypotonia and gait abnormalities are common, also consistent with animal model data^67^. Seizures were reported in 3/18 participants. Other neurodevelopmental concerns include ADHD, anxiety, Developmental Coordination Disorder, and mood disorders. Gastrointestinal distress and sleep dysfunction were also reported. Last, both dysmorphia and other organ anomalies were reported (conductive hearing loss- and coronary artery fistula). Within pairs of siblings sharing a variant, there is a similarity of phenotype, with some variability in the severity of the intellectual disability.

Different *de novo* mutations in *SHANK3* have also been associated with other developmental/neuropsychiatric disorders and genetic syndromes such as schizophrenia^47,68^ and Phelan-McDermid Syndrome (PMS)^69^. The majority of children diagnosed with PMS also have ASD, and both conditions are often associated with intellectual and language delay, hypotonia, seizures, and sleep disorders, although children with PMS also often have other organ involvement. We also examined the whole genomes from the MSSNG and SSC p.Ala1227Glyfs*69 carriers and assessed for other clinically relevant variants that could be contributing to the varying phenotypic presentation, but none were identified. Additionally, no other clinically relevant variants were highlighted in those individuals described in the literature^36,38,69–71^.

To evaluate if common genetic variants may be contributing to the ASD phenotype in the p.Ala1227Glyfs*69 *SHANK3* variant carriers, we calculated their ASD polygenic risk score (PRS) for all accessible individuals from European ancestry in MSSNG (db6) and SSC. PRS in the probands analyzed in this study varied between −1.167 and 15.606 (Table 2), showing no clear pattern between the presence of the clinically significant *SHANK3* variant and the polygenetic risk of common variants. PRS in all subjects with autism in MSSNG and SSC ranges between −18.580 and 20.626.

## Discussion

Our data indicate that 17/17 carriers (from 15 independent families) of the p.Ala1227Glyfs*69 variant affecting *SHANK3* who have been formally tested carry a diagnosis of ASD. Our analysis did not identify any other obvious rare or common genetic variants, or combinations thereof, in the genomes of these individuals that could be contributing to the phenotypes reported in these individuals. Given the nature of neurobehavioral complexity, perhaps not surprisingly, there is phenotypic heterogeneity exhibited amongst p.Ala1227Glyfs*69 carriers, which is a hallmark of autism^72,73^, as well as other related brain disorders that may share overlapping clinical features and contributory susceptibility genes^74,75^. It is instructive for future “genotype-first” queries that the discovery of this recurrent p.Ala1227Glyfs*69 variant was missed in our early analyses. It was only detected here upon careful consideration of the different naming schemes of the various isoforms (and exons within them) in *SHANK3*, which also varied between different software tools, as well as the various genome builds being compared against (Table 1)^76,77^.

In addition, we searched for p.Ala1227Glyfs*69 *SHANK3* variants in unpublished data from the SPARK cohort^41^. From 8744 ASD-affected individuals for which sequencing data from both parents were available, the variant was detected in two male individuals, both *de novo*. The variant was also detected in three out of 13,156 ASD-affected individuals (two males and one female) for which parental sequences were not available and thus inheritance could not be determined. As well from a private database we identified a female teen with ASD which based on the Vineland she would be described as severe, severe language delay, and severe global developmental delay. As highlighted on continuous measures of emotional difficulties (CBCL), she also presents with attention difficulties. This individual was not included in Table 3 since gold standard ASD measures were not available and this phenotype description is based on available assessments. We mention this data just to demonstrate that the variant is found in other collections, as would be expected, and await the presentation of more detailed phenotype data from these participants.

Two independently-created murine models with an insertion of a guanine nucleotide into the analogous mouse base pair position, which we refer to here as Shank3 InsG3680, have also demonstrated changes in cellular, circuit, and behavioral phenotypes^67,78^ (Supplementary Material; Table S2). Specifically, these Shank3InsG3680 mouse models demonstrated changes to baseline neurotransmission and/or impairments in long-term depression (LTD) and long-term potentiation (LTP), the synaptic basis of learning and memory. Overall homozygous Shank3InsG3680 +/+ mice exhibited more significant changes than heterozygous Shank3InsG3680 mice, suggesting that functioning of one normal Shank3 copy maybe sufficient to support some of its function.

Regional differences in synaptic deficits and synaptic composition were observed, and the extent of the impact may have been modulated by other Shank family genes. In the adult hippocampus, expression of the reversible Shank3InsG3680 variant cassette^67^ produced a truncated Shank3 protein and loss of the major high molecular weight isoforms at the synapse. This was associated with impaired hippocampal mGluR dependent LTD, intact LTP, and changes to baseline NMDA receptor (NMDAR) mediated synaptic function. In the striatum, Zhou et al.^78^ showed a significant decrease of levels of Shank3 mRNA in the Shank3InsG3680 strain compared with the wild type, suggesting a reduced level of mRNA through nonsense-mediated decay. This finding suggests that the InsG3680 variant results in a near-complete loss of SHANK3 protein, concomitant with synaptic transmission deficits in juvenile and adult homozygous mutant Shank3InsG3680 (+/+) mice. Post-translational modifications, modulated by nitric oxide, were also found in both young and adult Shank3InsG3680 +/+mice.

In assessments of general cognitive function, Shank3InsG3680 +/+ mice showed mild spatial learning impairments in the Morris Water Maze task and motor learning deficits in the accelerating rotarod task, while heterozygous mice did not^67^. ASD-associated behaviors in these two models also showed mixed outcomes in both social interaction impairments and repetitive behaviors that, similar to human assessments, may be dependent on age and gender. Speed et al.^67^ reported statistically different effects in some of their assessments comparing between male and female adult mice. This group did not observe social interaction deficits in the three-chamber task with mixed-sex adult mutant mice, nor did they observe repetitive behaviors, but instead suggested aversion to novel objects. However, in large all-male cohorts, Zhou et al.^78^ showed deficits in social behaviors in both juvenile and adult mice. In addition, in adults there was increased anxiety, repetitive grooming behaviors, and sensory processing differences^78^. On balance, the mouse data seems to generally recapitulate the learning impairments and behavioral differences seen in patients with the p.Ala1227Glyfs*69 *SHANK3* variant.

Highly penetrant alleles such as p.Ala1227Glyfs*69 in neurodevelopmental disorders are under severe negative selection and are constantly being removed from the population^79,80^. However, recurrent mutations are always being added to the gene pool and while typically occurring randomly, the intrinsic^81^ and extrinsic characteristics^82^ may also have an influence^83^. Experimental investigations have shown that guanine bases can be targets for oxidative damage in DNA, while mutability in other bases is more variable^84^. Moreover, the locus under study is within 8 guanines, which constitutes a homopolymer run (HR). HRs are sequences with six or more identical nucleotides and are associated with >10-fold enrichment of mutation compared to the genomic average^85^. It is noteworthy that there are three other G homopolymer runs in *SHANK3*, but no recurrent variants were found at these sites.

The CpG content of DNA has also been shown to influence the mutation rate in non-CpG-containing sequences, suggesting that intrinsic properties of DNA sequences may be more important than the chromosomal environment in determining mutation rates and genome integrity. Evidence indicates that because of the propensity for methyl-CpGs to deaminate and produce mismatches, it is plausible that error-prone repair mechanisms may have a role in hypermutability. CpG methylation might also have epigenetic effects by promoting chromatin states that make DNA more susceptible to mutations^86^.

Although exceedingly rare (0.075% frequency in the ASD families studied by WGS), the finding that this p.Ala1227Glyfs*69 variant in *SHANK3* is, so far, concordant with an ASD, and that it will surely continue to sporadically re-occur in the population, has important implications for genetic counseling. It will also be important to continue to search for the p.Ala1227Glyfs*69 variant in *SHANK3* to see if it confers risk in other disorders, including perhaps under a multiple-variant model^87^. Defining a specific mutational mechanism underlying an ASD outcome, may also focus strategies for the development of therapeutic interventions.

## Methods

### Genome sequence analysis

We searched ASD-specific genomic databases in which the participants upon recruitment had a diagnosis of ASD, for damaging *de novo* sequence-level variants affecting exactly the same genomic location in different families. A variant was defined to be damaging if it caused loss-of function (stop gain, frameshift, or canonical splice site-disrupting) or was a predicted deleterious missense variant based on American College of Medical Genetics guidelines^29^. Initially, we examined rare (frequency less than 0.001 in gnomAD and 1000 g) *de novo* variants identified from MSSNG data release DB6 (release date June 24, 2020), which were detected as previously described^6^. After identifying this recurrent variant in *SHANK3*, we then searched our in-house databases and performed literature searches for the same variant. Ethical review of these cohort studies was approved by institutional review boards and included assessing datasets through applications to Data Access Committees.

### Phenotyping measures

Phenotypic data was extracted either from the original manuscripts, in which case we attempted to stay close to the original descriptions or from the reference databases. In the latter case, clinical diagnosis of autism spectrum disorder was reported in the databases and was supported by ADI/ADOS. Intellectual disability was reported as a clinical diagnosis and in most cases formal IQ testing was available for confirmation. Language delay was available as a clinical diagnosis, often with characterizations, such as “minimally verbal” or “nonverbal” and in many cases formal language measure scores were available for review. Information on psychiatric/ neurological comorbidities was extracted from the original manuscripts, or available as a clinician diagnosis or clinical concern based on continuous measures of such symptomatology available (e.g., CBCL, RCADS).

### Confirming representation of exon 21 in exome and WGS datasets

Given the high GC-density content of *SHANK3*, which can influence exon capture and sequencing^52^, we thought it was critical when assessing mutational frequency to confirm that there were no biases in read-coverage of the site of the target variant within exon 21 (Supplementary Material; Fig.1). Using whole-exome sequences from 298 patients and 462 controls from our internal dataset, we ran the Agilent Sureselect Clinical research exome V1 for exome sequence analysis and show that the coverage around the G duplication region is at the anticipated 120x coverage (Supplementary Material; Fig. 1). This analysis also indicates that diagnostic exome sequencing will more than adequately capture and accurately genotype this position. WGS analysis of probands from MSSNG and SSC also confirm that exon 21 in *SHANK3* is uniformly covered.

### Protein and evolutionary conservation analysis

We used the DISOPRED3 predictor^49^ and the consensus of eight predictors from MobiDB-lite^50^ to map where the p.Ala1227Glyfs*69 variant falls with respect to intrinsically disordered regions (IDRs) of the protein. The variant isoforms were also analyzed using Feature Analysis of Intrinsically Disordered Regions^55,56^ and using PScore^57^. We analyzed the genomic conservation of the p.Ala1227Glyfs*69 variant with GERP^64^, UCSC PhyloP, and phastCons for primates, placental mammals, and 100 vertebrates^65^. The main text, tables, and figures (including Supplemental) have additional details relevant to the presentation of the results.

### Polygenic risk score analysis (PRS)

PRS was calculated for all individuals from European ancestry in MSSNG (db6) and SSC merged with 1000 Genomes European population using GWAS summary statistics derived from the iPSYCH Autism project including 13,076 cases and 22,664 controls from Denmark^88^. This included probands MSSNG00342-003, MSSNG0342-004, 1-1047-003, 2-1774-003, and 14470.p1. A total of 25,837 SNPs were included in PRS calculation. Since the proband 7-0527-003 was part of a later version of the MSSNG cohort (db7), he was not included in the initial PRS calculation. This individual’s PRS was calculated separately with his parents (7-0527-001 and 7-0527-002) using the same 25,837 SNPs included in PRS calculations for the others and centered by the mean in whole MSSNG/SSC/1000 Genomes European population. However, of 25,837 SNPs, 1496 were missing due to sample quality in this family, and caution is needed in comparison with the other subjects. The approach for interpretation of the PRS data was based on the previous studies^18,88,89^.

### Study recruitment

This study has complied with all relevant ethical regulations including obtaining informed consent from all participants and was approved by the Research Ethics Board at The Hospital for Sick Children.

### Reporting Summary

Further information on research design is available in the Nature Research Reporting Summary linked to this article.

## Supplementary information

Supplementary Information

Reporting Summary

## Data Availability

Access to the whole-genome sequence and phenotype information from MSSNG and SSC data can be obtained by completing data access agreements (https://research.mss.ng and https://www.sfari.org/resource/sfari-base, respectively), as was done for this study. These two well-established and stable whole-genome sequence and phenotype resources are utilized by approved investigators worldwide. The 1000 G genome-sequencing data are publicly available via Amazon Web Services (https://docs.opendata.aws/1000genomes/readme.html). Access to data through other publications or resources is described in the main text and is outlined in Table 2. Whole-genome sequence for 7-0572-003 will be available in the MSSNG database in its next release but can be requested in advance by contacting the corresponding author. The relevant variant information from the exome or direct Sanger sequencing data for the individuals for which whole-genome sequencing data does not exist and is described for the first time in this paper (HNDS_0130-01; 1505221080) is found in Table 2. Additional data can also be requested by contacting the corresponding author.

## References

[1] Tammimies K (2015). Molecular diagnostic yield of chromosomal microarray analysis and whole-exome sequencing in children with autism spectrum disorder. JAMA - J. Am. Med. Assoc..

[2] Fernandez BA, Scherer SW (2019). Syndromic autism spectrum disorders: moving from a clinically defined to a molecularly defined approach. Syndromic autism spectrum disorders - Fernandez and Scherer Dialogues in. Clin. Neurosci..

[3] Betancur C (2011). Etiological heterogeneity in autism spectrum disorders: more than 100 genetic and genomic disorders and still counting. Brain Res..

[4] Satterstrom FK (2020). Large-scale exome sequencing study implicates both developmental and functional changes in the neurobiology of autism. Cell.

[5] Bourgeron T (2015). From the genetic architecture to synaptic plasticity in autism spectrum disorder. Nat. Rev. Neurosci..

[6] Yuen RK (2017). Whole genome sequencing resource identifies 18 new candidate genes for autism spectrum disorder. Nat. Neurosci..

[7] Sanders SJ (2015). Insights into autism spectrum disorder genomic architecture and biology from 71 risk loci. Neuron.

[8] Woodbury-Smith M, Scherer SW (2018). Progress in the genetics of autism spectrum disorder. Developmental Med. Child Neurol..

[9] Vorstman JAS (2017). Autism genetics: opportunities and challenges for clinical translation. Nat. Rev. Genet..

[10] Srivastava S (2019). Meta-analysis and multidisciplinary consensus statement: exome sequencing is a first-tier clinical diagnostic test for individuals with neurodevelopmental disorders. Genet. Med..

[11] Schaaf CP (2020). A framework for an evidence-based gene list relevant to autism spectrum disorder. Nat. Rev. Genet..

[12] Hoang N, Buchanan JA, Scherer SW (2018). Heterogeneity in clinical sequencing tests marketed for autism spectrum disorders. npj Genomic. Medicine.

[13] Yehia L (2020). Copy number variation and clinical outcomes in patients with germline PTEN mutations. JAMA Netw. Open.

[14] Scherer SW, Dawson G (2011). Risk factors for autism: translating genomic discoveries into diagnostics. Hum. Genet..

[15] Anagnostou E (2018). Clinical trials in autism spectrum disorder: evidence, challenges and future directions. Curr. Opin. Neurol..

[16] Sahin M, Sur M (2015). Genes, circuits, and precision therapies for autism and related neurodevelopmental disorders. Science.

[17] Yuen RK (2015). Whole-genome sequencing of quartet families with autism spectrum disorder. Nat. Med..

[18] Leblond CS (2019). Both rare and common genetic variants contribute to autism in the Faroe Islands. npj Genom. Med..

[19] Simons Vip C (2012). Simons Variation in Individuals Project (Simons VIP): a genetics-first approach to studying autism spectrum and related neurodevelopmental disorders. Neuron.

[20] Miller DT (2010). Consensus statement: chromosomal microarray is a first-tier clinical diagnostic test for individuals with developmental disabilities or congenital anomalies. Am. J. Hum. Genet..

[21] Riggs ER (2020). Technical standards for the interpretation and reporting of constitutional copy-number variants: a joint consensus recommendation of the American College of Medical Genetics and Genomics (ACMG) and the Clinical Genome Resource (ClinGen). Genet Med.

[22] Pinto D (2014). Convergence of genes and cellular pathways dysregulated in autism spectrum disorders. Am. J. Hum. Genet..

[23] Merikangas AK (2015). The phenotypic manifestations of rare genic CNVs in autism spectrum disorder. Mol. Psychiatry.

[24] Malhotra D, Sebat J (2012). CNVs: harbingers of a rare variant revolution in psychiatric genetics. Cell.

[25] Marshall CR (2008). Structural variation of chromosomes in autism spectrum disorder. Am. J. Hum. Genet..

[26] Sanders SJ (2018). Progress in understanding and treating SCN2A-mediated disorders. Trends Neurosci..

[27] Frazier TW (2019). Autism spectrum disorder associated with germline heterozygous PTEN mutations. Cold Spring Harb. Perspect. Med..

[28] Bernier R (2014). Disruptive CHD8 mutations define a subtype of autism early in development. Cell.

[29] Richards S (2015). Standards and guidelines for the interpretation of sequence variants: a joint consensus recommendation of the American College of Medical Genetics and Genomics and the Association for Molecular Pathology. Genet Med.

[30] Jiang YH (2013). Detection of clinically relevant genetic variants in autism spectrum disorder by whole-genome sequencing. Am. J. Hum. Genet..

[31] Trost B (2020). Genome-wide detection of tandem DNA repeats that are expanded in autism. Nature.

[32] Fischbach GD, Lord C (2010). The simons simplex collection: a resource for identification of autism genetic risk factors. Neuron.

[33] Werling DM (2018). An analytical framework for whole-genome sequence association studies and its implications for autism spectrum disorder. Nat. Genet..

[34] Lord C, Cook EH, Leventhal BL, Amaral DG (2000). Autism spectrum disorders. Neuron.

[35] Rutter, M., LeCouteur, A. & Lord, C. *(ADI™-R) Autism Diagnostic Interview–Revised*. (WPS, 2003).

[36] Durand CM (2007). Mutations in the gene encoding the synaptic scaffolding protein SHANK3 are associated with autism spectrum disorders. Nat. Genet..

[37] Belmadani M (2019). VariCarta: A Comprehensive Database of Harmonized Genomic Variants Found in Autism Spectrum Disorder Sequencing Studies. Autism Res..

[38] De Rubeis S (2018). Delineation of the genetic and clinical spectrum of Phelan-McDermid syndrome caused by SHANK3 point mutations. Mol. Autism.

[39] Zhou WZ (2019). Targeted resequencing of 358 candidate genes for autism spectrum disorder in a Chinese cohort reveals diagnostic potential and genotype–phenotype correlations. Hum. Mutat..

[40] O’Roak BJ (2014). Recurrent de novo mutations implicate novel genes underlying simplex autism risk. Nat. Commun..

[41] Feliciano P (2019). Exome sequencing of 457 autism families recruited online provides evidence for autism risk genes. npj Genom. Med..

[42] Farwell KD (2015). Enhanced utility of family-centered diagnostic exome sequencing with inheritance model-based analysis: Results from 500 unselected families with undiagnosed genetic conditions. Genet. Med..

[43] Lim ET (2017). Rates, distribution and implications of postzygotic mosaic mutations in autism spectrum disorder. Nat. Neurosci..

[44] Ramu A (2013). DeNovoGear: De novo indel and point mutation discovery and phasing. Nat. Methods.

[45] Bidinosti M (2012). CLK2 inhibition ameliorates autistic features associated with SHANK3 deficiency. Scien.

[46] Gouder L (2019). Altered spinogenesis in iPSC-derived cortical neurons from patients with autism carrying de novo SHANK3 mutations. Sci. Rep..

[47] Gauthier J (2010). De novo mutations in the gene encoding the synaptic scaffolding protein SHANK3 in patients ascertained for schizophrenia. Proc. Natl Acad. Sci. USA.

[48] Durand CM (2012). SHANK3 mutations identified in autism lead to modification of dendritic spine morphology via an actin-dependent mechanism. Mol. Psychiatry.

[49] Jones DT, Cozzetto D (2015). DISOPRED3: Precise disordered region predictions with annotated protein-binding activity. Bioinformatics.

[50] Necci M, Piovesan D, Dosztanyi Z, Tosatto SCE (2017). MobiDB-lite: Fast and highly specific consensus prediction of intrinsic disorder in proteins. Bioinformatics.

[51] Csizmok V, Follis AV, Kriwacki RW, Kay JDF- (2017). Dynamic protein interaction networks and new structural paradigms in signaling. Physiol. Behav..

[52] Moessner R (2007). Contribution of SHANK3 mutations to autism spectrum disorder. Am. J. Hum. Genet..

[53] Zeng M (2016). Phase Transition in Postsynaptic Densities Underlies Formation of Synaptic Complexes and Synaptic Plasticity. Cell.

[54] Chen X, Wu X, Wu H, Zhang M (2020). Phase separation at the synapse. Nat. Neurosci..

[55] Zarin T (2019). Proteome-wide signatures of function in highly diverged intrinsically disordered regions. eLife.

[56] Zarin, T. et al. Identifying molecular features that are associated with biological function of intrinsically disordered protein regions. *bioRxiv*, 1–23, (2020). 10.7554/eLife.60220 PMC7932695 33616531

[57] Vernon RMC (2018). Pi-Pi contacts are an overlooked protein feature relevant to phase separation. eLife.

[58] Tsang B, Pritišanac I, Scherer SW, Moses AM, Forman-Kay JD (2020). Phase Separation as a Missing Mechanism for Interpretation of Disease Mutations. Cell.

[59] Karczewski KJ (2020). The mutational constraint spectrum quantified from variation in 141,456 humans. Nature.

[60] Phan, L., Jin, Y. & Zhang, Z. ALFA: Allele Frequency Aggregator. National Center for Biotechnology Information, U.S. National Library of Medicine (2020).

[61] Taliun D (2021). Sequencing of 53,831 diverse genomes from the NHLBI TOPMed Program. Nature.

[62] Reuter MS (2018). The Personal Genome Project Canada: findings from whole genome sequences of the inaugural 56 participants. CMAJ.

[63] Pinese M (2020). The Medical Genome Reference Bank contains whole genome and phenotype data of 2570 healthy elderly. Nat. Commun..

[64] Davydov EV (2010). Identifying a high fraction of the human genome to be under selective constraint using GERP. PLoS Comput. Biol..

[65] Kuhn RM, Haussler D, James Kent W (2013). The UCSC genome browser and associated tools. Brief. Bioinforma..

[66] Henn BM (2016). Distance from sub-Saharan Africa predicts mutational load in diverse human genomes. Proc. Natl Acad. Sci. USA.

[67] Speed HE (2015). Autism-associated insertion mutation (InsG) of shank3 exon 21 causes impaired synaptic transmission and behavioral deficits. J. Neurosci..

[68] De Sena Cortabitarte A (2017). Investigation of SHANK3 in schizophrenia. Am. J. Med. Genet., Part B: Neuropsychiatr. Genet..

[69] Leblond CS (2014). Meta-analysis of SHANK mutations in autism spectrum disorders: a gradient of severity in cognitive impairments. PLoS Genet..

[70] Bonaglia MC (2001). Disruption of the ProSAP2 gene in a t(12;22)(q24.1;q13.3) is associated with the 22q13.3 deletion syndrome. Am. J. Hum. Genet..

[71] Du X (2018). Genetic diagnostic evaluation of trio-based whole exome sequencing among children with Diagnosed or suspected autism spectrum disorder. Front. Genet..

[72] Pelphrey KA, Shultz S, Hudac CM, Vander Wyk BC, Manuscript A (2012). Development in autism spectrum disorder. J. Child Psychol. Psychiatry.

[73] Castelbaum L, Sylvester CM, Zhang Y, Yu Q, Constantino JN (2020). On the nature of monozygotic twin concordance and discordance for autistic trait severity: a quantitative analysis. Behav. Genet..

[74] Myers SM (2020). Insufficient Evidence for “Autism-Specific” Genes. Am. J. Hum. Genet..

[75] State MW, Levitt P (2011). The conundrums of understanding genetic risks for autism spectrum disorders. Nat. Neurosci..

[76] Bruford EA (2020). Guidelines for human gene nomenclature. Nat. Genet.

[77] Stenson PD (2020). The Human Gene Mutation Database (HGMD((R))): optimizing its use in a clinical diagnostic or research setting. Hum. Genet.

[78] Zhou Y (2016). Mice with Shank3 Mutations Associated with ASD and Schizophrenia Display Both Shared and Distinct Defects. Neuron.

[79] Uher R (2009). The role of genetic variation in the causation of mental illness: an evolution-informed framework. Mol. Psychiatry.

[80] Yuen RK (2016). Genome-wide characteristics of de novo mutations in autism. NPJ Genom. Med.

[81] Ellegren H, Smith NGC, Webster MT (2003). Mutation rate variation in the mammalian genome. Curr. Opin. Genet. Dev..

[82] Crow JF (2000). The origins, patterns and implications of human spontaneous mutation. Nat. Rev. Genet..

[83] Michaelson JJ (2012). Whole-genome sequencing in autism identifies hot spots for de novo germline mutation. Cell.

[84] Růžička M (2017). DNA mutation motifs in the genes associated with inherited diseases. PLoS ONE.

[85] Montgomery SB (2013). The origin, evolution, and functional impact of short insertion-deletion variants identified in 179 human genomes. Genome Res.

[86] Swami M (2010). Mutation: It’s the CpG content that counts. Nat. Rev. Genet..

[87] Leblond CS (2012). Genetic and functional analyses of SHANK2 mutations suggest a multiple hit model of autism spectrum disorders. PLoS Genet..

[88] Grove J (2019). Identification of common genetic risk variants for autism spectrum disorder HHS Public Access Author manuscript. Nat. Genet..

[89] D’Abate L (2019). Predictive impact of rare genomic copy number variations in siblings of individuals with autism spectrum disorders. Nat. Commun..

