# Supplementary material for: A recurrent SHANK3 frameshift variant in Autism Spectrum Disorder
Source: NPJ Genom Med. 2021 Nov 4;6:91. doi: 10.1038/s41525-021-00254-0 (PMC8568906; doi:10.1038/s41525-021-00254-0)
Supplement: Supplementary file 1 — Supplementary Information [file 41525_2021_254_MOESM1_ESM.pdf]

SUPPLEMENTARY INFORMATION

Supplementary Table 1 – Recurrent *de novo* damaging missense variants found in ASD probands in the MSSNG and SSC cohorts. Variants frequencies are less than 0.001 in gnomAD and 1000g.

Gene	CHR	Start	End	Reference bases	Alternate bases	Protein Change	Sample ID	Sex
PTEN	chr10	87957940	87957941	T	G	p.F241L	2-1223-003	F
							7-0250-003	M
CSNK1E	chr22	38300756	38300757	G	A	p.R178C	5-5202-003	F
							REACH000519	F
CAMK2A	chr5	150251807	150251808	G	A	p.P212L	AU2306301	M
							REACH000664	M
SPTAN1	chr9	128632636	128632637	T	C	c.T7142C	AU2564301	M
							MSSNG00044-003	F
MECP2	chrX	154030911	154030912	G	A	p.R213C	MSSNG00370-003	F
							MT_75.3	F

Supplementary Table 2- Comparison between the phenotypes observed in two mouse models engineered to carry the murine equivalent of the *SHANK3* p.Ala1227Glyfs*69 variant found in humans. Phenotypes in humans are described in Table2.

Phenotype	Mouse - *Shank3G* exon 21<br>Speed et al.[1]	Mouse - InsG3680<br>Zhou et al.[2]	Humans (Table 2)
Intellectual disability	x	x	x
Impaired motor coordination	x	x	x
Altered response to novelty	X (object)	x	
Anxiety-like behaviour		x	x
Social interaction deficits		x	x
Repetitive/compulsive behaviour		x	

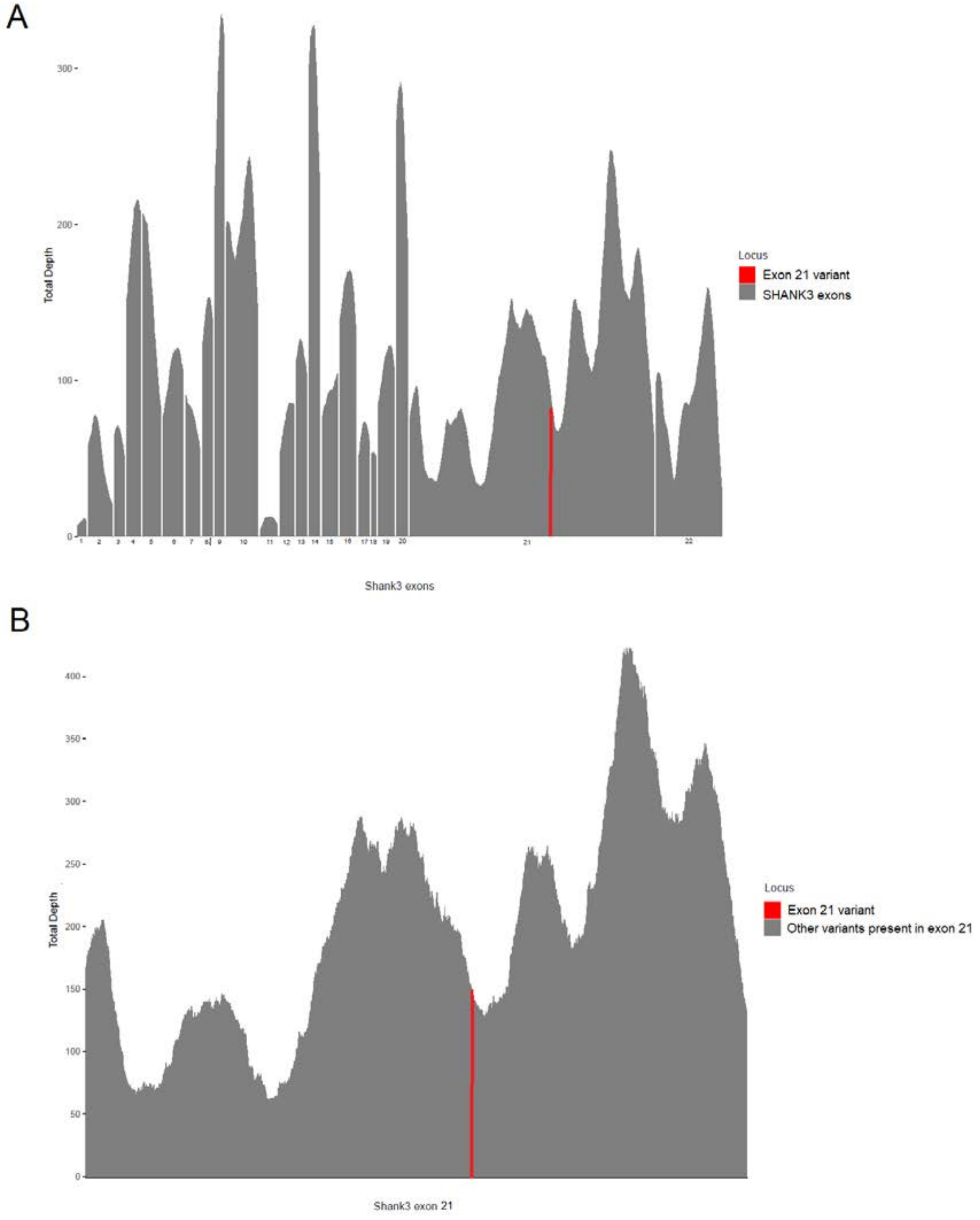

Supplementary Figure 1 - A – *SHANK3* exon coverage per base pair calculated for exon 1 to 22 in 698 individuals. B – *SHANK3* exon 21 coverage calculated for 462 individuals. Red line indicates the position of the guanine duplication described in this study. Whole Genome sequencing also indicates high coverage sequencing across *SHANK3*, including in exon 21.

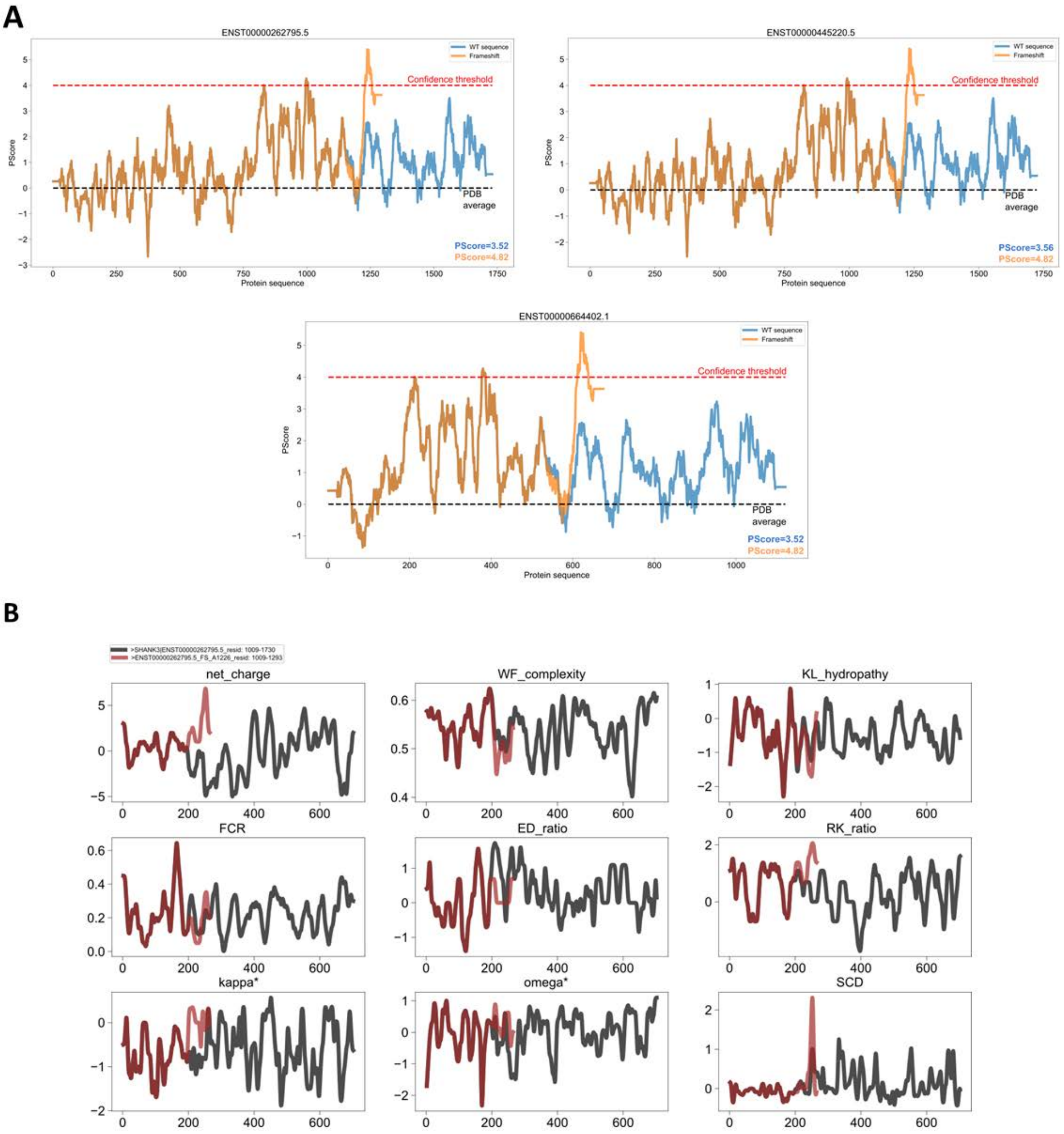

Supplementary Figure 2. A- Sequence profiles of PScore[3], a predictor of phase separation via planar pi-pi interactions in intrinsically disordered proteins, for each of the three isoforms and the corresponding variant; B- Sequence profiles from FAIDR[4] for the isoform and the corresponding variant for the isoform ENST00000262795.5, highlighting different physicochemical features known to be associated with phase separation, including ratios of arginine to lysine (RK ratio) and two measures of charge patterning (kappa and sequence charge decoration, SCD). See Zarin et al.[5] for more details on these features.

Warning: This report is based on knowledge and data that are not firmly established. Consequently, medical decisions must not be made on the basis of this report.

SHANK3 (SH3 and multiple ankyrin repeat domains 3) Variation

Duplication (1 bp) in exon 24.
This variation creates a frame shift starting at codon Ala1226. The new reading frame ends in a STOP codon at position 69.

This variant is known to ClinVar (February-2021): RCV000004730.7 (Pathogenic** - 22q13.3 deletion syndrome), RCV000190779.1 (Pathogenic* - Inborn genetic diseases), RCV000366708.2 (Pathogenic** - not provided), RCV000754675.1 (Pathogenic* - Autism spectrum disorder), RCV000719974.1 (Pathogenic* - History of neurodevelopmental disorder).

This variant is known to dbSNP (151): rs797044936 (validated dbSNP entry - Clinical significance: CLIN_pathogenic).

This variant is known to ESP (ESP6500SIV2): Eur. Am.: TGG=1.30% - Afr. Am.: TGG=1.01%

This variant is known to gnomAD (2.1) <Exomes>: ALL:0.0099% - AMR:0.0079% - ASJ:0.012% - SAS:0.0084% - NFE:0.014% - FIN:0.013% (**Filter**: RF)

HGVS Nomenclature (v15.11)

---

cDNA Level: **ENST00000262795.5:c.3676dup**
gDNA Level: **Chr22(GRCh38):g.50721512dup**
Protein Level: **p.(Ala1226Glyfs*69)**

©Interactive Biosoftware - Created by Alamut Visual v.2.15.0 on 2021-03-19

Supplementary Figure 3. The output of the Alamut Visual version 2.15 (SOPHiA GENETICS, Lausanne, Switzerland) annotation tool when indicating a duplication of any one of the guanines in the Chr22(GRCh38:)g.50,721,505-50,721,512 region. Regardless of which G is selected in this homopolymer run as duplicated the variant is annotated as a duplication of the final G at the 3' end of the gene according to standard HGVS nomenclature (https://varnomen.hgvs.org/). The high frequency of the variant reported in ESP is likely to be artifactual since coverage at this position is low in the ESP database and the frequency is low in newer population databases like gnomAD. Moreover, even in gnomAD the variant is only present in the filtered reads, suggesting low variant quality.

SUPPLEMENTARY REFERENCES

1 Speed, H. E., Kouser, M., Xuan, Z., Reimers, J. M., Ochoa, C. F. *et al.* Autism-Associated Insertion Mutation (InsG) of Shank3 Exon 21 Causes Impaired Synaptic Transmission and Behavioral Deficits. *J Neurosci* **35**, 9648-9665, (2015).

2 Zhou, Y., Kaiser, T., Monteiro, P., Zhang, X., Van der Goes, M. S. *et al.* Mice with Shank3 Mutations Associated with ASD and Schizophrenia Display Both Shared and Distinct Defects. *Neuron* **89**, 147-162, (2016).

3 Vernon, R. M., Chong, P. A., Tsang, B., Kim, T. H., Bah, A. *et al.* Pi-Pi contacts are an overlooked protein feature relevant to phase separation. *Elife* **7** (2018).

4 Zarin, T., Strome, B., Peng, G., Pritišanac, I., Forman-Kay, J. D. *et al.* Identifying molecular features that are associated with biological function of intrinsically disordered protein regions. *bioRxiv*, 1-23, (2020).

5 Zarin, T., Strome, B., Nguyen Ba, A. N., Alberti, S., Forman-Kay, J. D. *et al.* Proteome-wide signatures of function in highly diverged intrinsically disordered regions. *eLife* **8**, 1-26, (2019).
